# Nurse-Led Educational Intervention Improves Self-Care Knowledge in Type 2 Diabetes: A Pre-Post Study Using the Diabetes Knowledge Questionnaire

**DOI:** 10.7759/cureus.87477

**Published:** 2025-07-07

**Authors:** Victor M Ayuso-Diaz, Jonathan J Colli-Garcia, Michelle A Hernandez-Pat

**Affiliations:** 1 Division of Research and Education, Medical Care and Research Center, Yucatán, MEX; 2 Genomic-Metabolic Unit, Marista University of Merida, Yucatán, MEX; 3 Department of Nursing, Universidad Autónoma de Campeche, Campeche, MEX

**Keywords:** chronic disease management, diabetes education, dkq-24, health literacy, latin american population, nursing intervention, patient empowerment, pre-post study, self-care knowledge

## Abstract

Objective

Type 2 diabetes mellitus (T2DM) is a chronic, non-communicable disease that is becoming increasingly prevalent around the world, particularly in low- and middle-income countries. Patient education plays a critical role in promoting the self-care behaviours that are essential for glycemic control and preventing complications. The aim of this study was to evaluate the effectiveness of a nurse-led educational intervention in improving diabetes knowledge in adults with T2DM, as measured by the Diabetes Knowledge Questionnaire (DKQ-24).

Materials and methods

A quasi-experimental pre-post study was conducted at the Medical Care and Research Centre in Mérida, Yucatán, Mexico, from April to May 2025. Thirty adult patients with confirmed T2DM and no prior structured diabetes education participated in a three-session educational programme delivered by trained nurses. The DKQ-24 was administered before and after the intervention. Descriptive statistics and paired t-tests were used to analyse changes in total and domain-specific knowledge scores. A Shapiro-Wilk test confirmed normality, with a significance level of p < 0.05 applied.

Results

The mean DKQ-24 score increased significantly from 24.1 ± 5.8 to 26.4 ± 4.5 (p = 0.0039). Notable improvements were seen in items addressing misconceptions about special diets, wound care, and insulin production. The most significant knowledge gains occurred in the disease control and complications domain. Nine out of 24 items showed statistically significant improvement post-intervention.

Conclusion

A brief educational intervention led by nurses significantly improved patients’ knowledge of diabetes, particularly by dispelling persistent myths. These findings highlight the important role of nurses in therapeutic education and support the incorporation of structured educational programmes into clinical diabetes care.

## Introduction

Type 2 diabetes mellitus (T2DM) is a chronic non-communicable disease (NCD) with high prevalence and insidious progression. It is currently considered one of the main threats to global public health [1]. According to global projections, it is estimated that more than 640 million adults will be living with diabetes by 2030, a figure expected to exceed 780 million by 2045 [2]. In Mexico, T2DM is the second leading cause of mortality and a major cause of disability, placing a significant clinical and economic burden on the healthcare system [3].

From a pathophysiological perspective, T2DM is characterized by a combination of peripheral insulin resistance and progressive pancreatic β-cell dysfunction, leading to chronic hyperglycaemia. This altered metabolic state promotes the development of microvascular complications such as retinopathy, nephropathy, and neuropathy, as well as macrovascular complications such as ischemic heart disease and cerebrovascular disease, which progressively deteriorate quality of life and increase the risk of premature mortality [4].

In regions such as Yucatán, the prevalence of T2DM exceeds the national average, according to the 2021 National Health and Nutrition Survey (ENSANUT). Sociocultural factors, limited access to healthcare services, and low health literacy levels exacerbate patient vulnerability in these regions [5]. The city of Mérida, in particular, faces significant challenges in providing comprehensive care for people with chronic diseases, especially those who have recently been diagnosed or have not previously participated in formal educational programmes [6,7].

Self-care is recognized as an essential component of T2DM treatment. This involves making informed decisions and taking action to maintain health and control disease: planning a diet, exercising regularly, monitoring blood sugar levels, taking medication as prescribed, and preventing complications. However, several studies have highlighted that knowledge of these practices is often inadequate among people with T2DM, thereby compromising their capacity for self-management [8].

In this context, therapeutic diabetes education led by nursing professionals has proven to be an effective strategy for improving knowledge, modifying behaviours, and strengthening self-management [8]. Due to their proximity to patients, understanding of the biopsychosocial environment, and ability to adapt educational messages according to cultural and cognitive levels, nurses are a strategic pillar in the implementation of health education programmes [6].

To evaluate the impact of such interventions, it is essential to use validated tools. The Diabetes Knowledge Questionnaire (DKQ-24) is a reliable instrument for measuring general diabetes knowledge and identifying specific areas for improvement in key topics such as nutrition, treatment, monitoring, and prevention of complications. Its validation in Spanish and its frequent use in Latin American studies support its applicability in clinical contexts such as Mexico [9,10].

In light of the above, the present study aims to evaluate the efficacy of a nurse-led educational intervention on self-care knowledge in patients with T2DM, by administering the DKQ-24 questionnaire before and after the intervention. The study was conducted at the Medical Care and Research Clinical Research Centre in Mérida, Yucatán, and focused on patients with no previous experience of structured diabetes education programmes. It is hoped that the findings will provide valuable evidence to reinforce the important role of nurses in the therapeutic education of chronically ill patients.

## Materials and methods

This pre-post quasi-experimental study, which did not include a control group, was designed to evaluate the impact of a nurse-led educational intervention on the self-care knowledge of patients with T2DM. The study population consisted of adult patients with a confirmed diagnosis of T2DM who regularly attended the Medical Care and Research Clinical Research Centre in Mérida, Yucatán, Mexico.

Inclusion criteria were: age 18 years or older, functional literacy (ability to read and write), no previous exposure to structured diabetes education programmes (i.e. naïve to such programmes), and provision of signed informed consent. Patients with documented cognitive impairment or physical or mental conditions that could limit their ability to actively participate were excluded.

Participants were consecutively recruited during routine outpatient consultations between April and May 2025. Eligible individuals were invited to participate, and after receiving a detailed explanation of the study, all provided written informed consent. The research was authorised and supported by Medical Care and Research and was conducted in accordance with the principles of beneficence, non-maleficence, respect for autonomy, and confidentiality, ensuring compliance with the Declaration of Helsinki and national regulations on research involving human participants.

The educational intervention, designed using adult learning principles, consisted of a single intensive 60-minute face-to-face session delivered by nurses trained in diabetes education. The session covered key aspects of T2DM self-care, including understanding the disease and its pathophysiology, pharmacological and non-pharmacological treatment options, healthy eating, glycaemic self-monitoring, prevention of complications, and recognition of warning signs. A participatory-expository methodology was employed, supported by printed and audiovisual materials adapted to the participants' literacy levels.

The DKQ-24 was used to assess knowledge, with the questionnaire administered immediately before and after the intervention to measure short-term knowledge acquisition. The DKQ-24 is a validated instrument for Spanish-speaking populations, with an internal consistency of α = 0.78 [10]. Each item offers three response options: 'Yes', 'No', and 'Don't know'. For analysis, correct responses were coded as 1, while incorrect or unknown responses were coded as 0. Permission to reproduce the DKQ-24 for this academic, non-commercial use was formally obtained from the original publisher (John Wiley & Sons) via RightsLink.

Scores for each domain (general knowledge, disease control, and complications) were calculated by summing the number of correct responses. Higher scores indicated greater knowledge of diabetes.

Statistical analysis was conducted using Stata version 14. Descriptive statistics (mean, SD, and range) were used to summarise sociodemographic variables and DKQ-24 scores. The Shapiro-Wilk test was used to assess the normality of differences. Paired t-tests were applied to compare pre- and post-intervention scores. A two-tailed p-value of less than 0.05 was considered statistically significant.

## Results

A total of 30 adult patients with a confirmed diagnosis of T2DM were enrolled in the study. The mean age of the participants was 56.3 ± 10.2 years, with an age range of 34-72 years. There was a slight predominance of female participants (n = 17; 56.7%), consistent with the sex distribution often observed in outpatient diabetes care settings [11]. This demographic composition provided a representative sample of the typical adult population receiving follow-up for T2DM at the participating clinical centre.

The 24 questions of the DKQ-24 were categorised under four themes: general diabetes information, disease control, complications, and overall knowledge. Analysis by domain revealed significant improvements in two areas, while the others showed non-statistically significant increases (Table 1).

**Table 1:** Comparison of mean DKQ‑24 scores before and after the nurse-led educational intervention by thematic domain (n = 30). Paired Student’s t-test was used for analysis. Scores were calculated using the original DKQ‑24 instrument developed by García AA et al. [10]. All items were reproduced in their original form with permission from John Wiley and Sons (License number: 6051370006501) for academic, non-commercial research purposes. DKQ: Diabetes Knowledge Questionnaire.

DKQ-24 Domain	Pre Mean ± SD	Post Mean ± SD	Mean Difference ± SD	95% CI (Lower-Upper)	t-value	p-value
General knowledge about diabetes	6.00 ± 2.01	6.23 ± 1.28	-0.23 ± 1.71	-0.87 to 0.41	-0.74	0.462
Knowledge about disease control	6.03 ± 1.83	7.03 ± 1.56	-1.00 ± 1.49	-1.55 to -0.44	-3.78	0.0009
Knowledge about complications	5.87 ± 1.57	6.73 ± 1.66	-0.87 ± 1.68	-1.49 to -0.24	-2.86	0.0083
Overall knowledge (all categories)	6.20 ± 1.54	6.43 ± 1.14	-0.23 ± 1.22	-0.69 to 0.22	-1.05	0.304

In the General Information domain, which covered basic disease concepts, the mean pre-intervention score was 6.00 ± 2.01, rising slightly to 6.23 ± 1.28 after the educational intervention, but this change did not reach statistical significance (mean difference: 0.23 ± 1.71; p = 0.462). By contrast, the Disease Control domain, which covers glycaemic monitoring, exercise, and treatment adherence, showed significant improvement, with a mean score increase from 6.03 ± 1.83 to 7.03 ± 1.56 (mean difference: 1.00 ± 1.49; p = 0.0009).

Significant progress was also observed in the Complications domain, which assessed aspects related to organ damage and the prevention of complications. The mean score increased from 5.87 ± 1.57 to 6.73 ± 1.66 (mean difference: 0.87 ± 1.68; p = 0.0083). In the Global Knowledge domain, which integrates all questionnaire categories, the mean score increased from 6.20 ± 1.54 to 6.43 ± 1.14, but this change was not statistically significant (mean difference: 0.23 ± 1.22; p = 0.304).

As shown in Table 1, these findings suggest that the educational intervention was particularly effective in strengthening practical knowledge related to diabetes control and complication prevention, rather than in enhancing general conceptual understanding of the disease.

Furthermore, analysis of individual items revealed a statistically significant improvement in nine out of 24 questions (37.5%), as determined by a paired Student’s t-test (p < 0.05). Notable improvements were observed in responses to questions concerning diabetes types (Q11), the consequences of hypoglycaemia (Q12), the choice between medication and lifestyle interventions (Q13), foot care and wound management (Q16 and Q17), and the use of appropriate compression garments (Q23). These results suggest that the intervention effectively addressed less intuitive or commonly misunderstood areas of diabetes management, such as differentiating between diabetes types, recognising hypoglycaemia, employing non-pharmacological strategies, and specific foot care practices.

In contrast, items addressing well-known or widely understood concepts, such as the relationship between sugar intake and diabetes (Q1), the chronic nature of the disease (Q5), and typical glucose level thresholds (Q8), did not show statistically significant improvement, possibly due to participants having a higher baseline level of knowledge in these areas (Table 2).

**Table 2:** Item-by-item mean differences (post-pre) in DKQ-24 scores among patients with type 2 diabetes mellitus (n = 30). Paired Student’s t-test was used for analysis. All item wording corresponds to the original DKQ-24 developed by García AA et al. [10], and was reproduced with permission from John Wiley and Sons (License number: 6051370006501), for academic and non-commercial research purposes. DKQ: Diabetes Knowledge Questionnaire.

Item	DKQ-24 Question	Mean ± SD	95% CI Lower	95% CI Upper	t-value	p-value
1	Eating too much sugar and other sweet foods is a cause of diabetes.	0.10 ± 0.15	-0.01	0.21	1.81	0.083
2	The usual cause of diabetes is lack of effective insulin in the body.	-0.20 ± 0.14	-0.48	0.08	-1.43	0.161
3	Diabetes is caused by failure of the kidneys to keep sugar out of the urine.	0.00 ± 0.11	-0.22	0.22	0	1
4	The kidneys produce insulin.	-0.03 ± 0.11	-0.26	0.2	-0.34	0.769
5	In untreated diabetes, the amount of sugar in the blood usually increases.	0.00 ± 0.10	-0.1	0.1	0	1
6	If I am diabetic, my children have a higher chance of being diabetic.	-0.10 ± 0.12	-0.25	0.05	-1.38	0.184
7	Diabetes can be cured.	0.07 ± 0.10	-0.13	0.26	0.7	0.489
8	A fasting blood sugar level of 210 is too high.	-0.03 ± 0.11	-0.15	0.09	-0.57	0.573
9	The best way to check my diabetes is by testing my urine.	-0.13 ± 0.14	-0.32	0.06	-1.45	0.161
10	Regular exercise will increase the need for insulin or other diabetic medication.	-0.13 ± 0.13	-0.3	0.03	-1.67	0.103
11	There are two main types of diabetes: Type 1 (insulin-dependent) and Type 2 (non-insulin-dependent).	-0.37 ± 0.14	-0.57	-0.16	-3.95	0.001
12	An insulin reaction is caused by too much food.	-0.40 ± 0.14	-0.69	-0.11	-3.62	0.008
13	Medication is more important than diet and exercise to control my diabetes.	-0.33 ± 0.12	-0.58	-0.09	-3.38	0.01
14	Diabetes often causes poor circulation.	-0.03 ± 0.11	-0.15	0.09	-0.57	0.573
15	Cuts and abrasions on diabetics heal more slowly.	0.07 ± 0.12	-0.17	0.31	0.57	0.573
16	Diabetics should take extra care when cutting their toenails.	-0.17 ± 0.11	-0.31	-0.03	-2.45	0.023
17	A person with diabetes should cleanse a cut with iodine and alcohol.	-0.23 ± 0.13	-0.45	-0.02	-2.3	0.032
18	The way I prepare my food is as important as the foods I eat.	-0.17 ± 0.12	-0.31	-0.03	-2.45	0.023
19	Diabetes can damage my kidneys.	0.03 ± 0.13	-0.28	0.35	0.21	0.832
20	Diabetes can cause loss of feeling in my hands, fingers, and feet.	0.00 ± 0.12	-0.2	0.2	0	1
21	Shaking and sweating are signs of high blood sugar.	-0.10 ± 0.13	-0.21	0.01	-1.79	0.083
22	Frequent urination and thirst are signs of low blood sugar.	0.00 ± 0.00	0	0	–	–
23	Tight elastic hose or socks are not bad for diabetics.	-0.20 ± 0.14	-0.38	-0.02	-2.32	0.031
24	A diabetic diet consists mostly of special foods.	0.03 ± 0.12	-0.12	0.19	0.44	0.662

**Figure 1:**
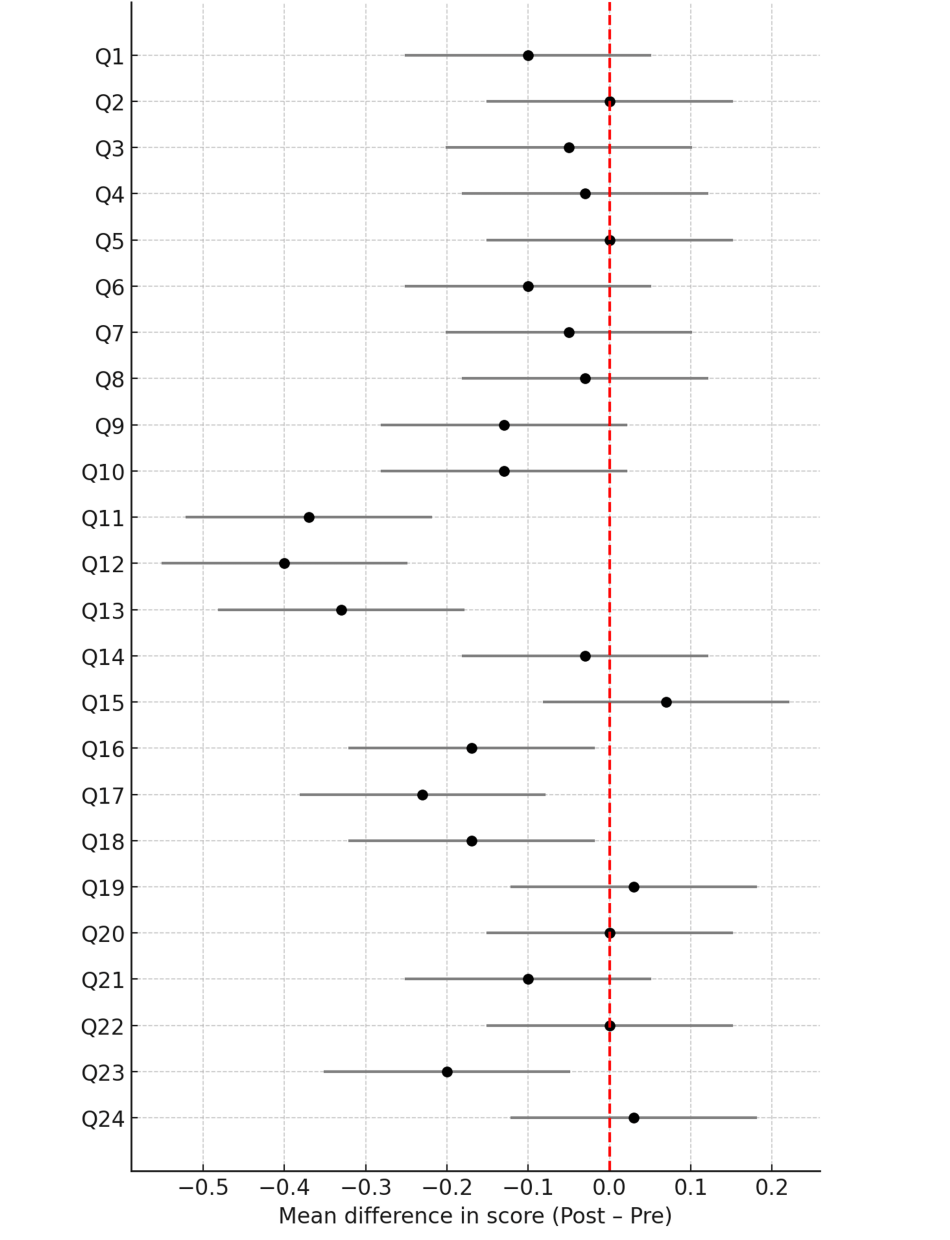
Forest plot of individual item changes in DKQ-24 scores (pre-post intervention). X-axis label: mean difference in score (post-pre). The plot displays the mean difference in scores for each DKQ-24 item (Q1-Q24), along with corresponding 95% CIs. The red vertical line represents no change (mean difference = 0). Points entirely to the left of the line indicate statistically significant improvement following the intervention. Q = Question number from the DKQ-24 questionnaire. DKQ: Diabetes Knowledge Questionnaire.

The complete results of this analysis are visualised in Figure 1, which presents a forest plot illustrating the magnitude and direction of changes for each question in a comparative format.

## Discussion

T2DM remains one of the leading contributors to the global health burden, particularly in middle-income countries such as Mexico [12]. In this context, factors such as low health literacy, cultural misconceptions, and limited access to continuous educational programmes converge to hinder effective self-management. Against this backdrop, our study provides evidence on the efficacy of a brief, nurse-led educational intervention for adult patients with no prior exposure to structured diabetes education [13].

In our cohort, mean scores increased from 24.1 ± 5.8 to 26.4 ± 4.5 after the intervention (p = 0.0039). The most notable improvements were observed in questions addressing widespread myths, such as the need for special diets, insulin misconceptions, and wound care. These findings suggest that certain misconceptions remain deeply rooted in this population, similar to those identified by Peña-Purcell NC et al. (2011) in Hispanic patients in the United States, where patient empowerment was key to challenging misinformation and modifying behaviours [14].

The 24 questions of the DKQ-24 were organised into four thematic domains. The greatest improvements were seen in the disease control domain (mean difference: -1.00 ± 1.49; p = 0.0009) and the knowledge of complications domain (mean difference: -0.87 ± 1.68; p = 0.0083). These results align with those of Velázquez López L et al. (2023), who observed that improvements are more likely in domains related to patients’ clinical or experiential knowledge, compared to abstract or conceptual domains that require deeper cognitive processing [15].

Similarly, García AA et al. (2001) demonstrated that culturally adapted interventions, delivered in the patient's native language and grounded in everyday life, consistently yielded significant improvements in diabetes-related knowledge. These gains were particularly evident when the educational content was reinforced through practical, relatable examples [10].

Despite consisting of only a single 60-minute session, our intervention was capable of generating measurable and meaningful improvements, particularly among patients who had never participated in formal diabetes education. Similar outcomes have been reported in brief interventions conducted in other Spanish-speaking countries, including Colombia, Spain, and the United States [9-14].

However, as emphasised by the Lancet Commission on Diabetes, the benefits of educational interventions often diminish over time if they are not embedded within broader, ongoing strategies that include structured follow-up, community involvement, or reinforcement mechanisms [14,15]. This underscores the importance of integrating such interventions into a long-term educational continuum, rather than treating them as isolated events.

Methodologically, the DKQ-24 proved useful for identifying cognitive gaps and tracking improvements. Nevertheless, the instrument showed limitations in addressing emerging dimensions of diabetes care, such as digital self-monitoring tools, new therapeutic approaches, and the role of technology-assisted interventions. As suggested by recent literature, updating or supplementing the questionnaire could enhance its relevance to contemporary diabetes management [9].

Finally, our findings highlight the pivotal role of nursing professionals in therapeutic education. Their proximity to patients, deep understanding of psychosocial contexts, and ability to adapt health messages to various literacy levels position them as key agents of change. This has been consistently supported by evidence from diverse Spanish-speaking populations [14-16]. 

This study has several limitations. Firstly, the quasi-experimental pre-post design without a control group limits the ability to draw strong causal inferences. However, this design was deemed appropriate for the setting due to feasibility constraints and the inherent challenges of ensuring baseline comparability between control and intervention groups, particularly with respect to differences in health literacy or cognitive capacity. Secondly, the small sample size, coupled with recruitment from a single clinical research centre in Mérida, may restrict the generalisability of the findings to broader or more diverse populations. Thirdly, the short-term nature of the evaluation prevented the assessment of knowledge retention over time. Finally, although the DKQ-24 is a validated instrument, it may not capture the latest developments in diabetes care, such as mobile technologies, digital monitoring tools, or advances in pharmacological therapy. Future studies should consider incorporating a control group where feasible, expanding the sample size, and recruiting participants from other regions. Long-term follow-up assessments should also be conducted to strengthen the evidence base.

## Conclusions

Our study found that a nurse-led educational intervention significantly improved self-care knowledge in patients with T2DM, as evidenced by statistically significant increases in DKQ-24 scores post-intervention. The most substantial gains were noted in items addressing persistent myths and incorrect practices, underscoring the importance of reinforcing health literacy through culturally tailored strategies. These findings highlight the critical role of nurses as strategic educational agents capable of transforming patients’ understanding of their condition and promoting sustainable self-care behaviours.
